# Proactive versus standard support of labour in nulliparous women; study protocol for a randomized, controlled trial

**DOI:** 10.1186/s13063-020-4191-9

**Published:** 2020-04-23

**Authors:** Møyfrid Brenne Fehn, Raija Dahlø, Renate Nielsen, Ingebjørg Laache, Eszter Vanky

**Affiliations:** 1 Department of Obstetrics and Gynecology, St. Olav’s Hospital, Trondheim University Hospital, Trondheim, Norway; 2 Department of Public Health and Nursing, Faculty of Medicine and Health Sciences, Norwegian University of Science and Technology, Trondheim, Norway; 3 Department of Clinical and Molecular Medicine, Faculty of Medicine and Health Sciences, Norwegian University of Science and Technology, Trondheim, Norway

**Keywords:** Nulliparous, Latent phase, Prolonged labour, Proactive support of labour

## Abstract

**Background:**

Prolonged latent phase of labour often results in a traumatic birth experience. Prolonged labour is associated with more operative deliveries, haemorrhage, fetal asphyxia and poor birth experience. Women with prolonged labour in a former pregnancy more often demand caesarean section in the next, due to their negative birth experience. *“*Proactive support of labour” is an alternative method, developed to counteract prolonged labour. There are little research and no randomized controlled study that compare *proactive* to *standard* labour support.

**Methods/Design:**

A prospective, non-blinded, randomized, single-centre, clinical trial where we compare proactive support to standard support of labour in a university hospital setting.

Inclusion criteria: latent phase of labour, non-pathologic pregnancy. Robson group 1, with painful contractions, and fully effaced cervix, with 1–3 cm dilatation. Exclusion criteria: induction of labour, breech presentation, twin pregnancy, multi-parity, conditions that require extended surveillance before and/or during labour.

Primary outcome: spontaneous, uncomplicated vaginal delivery. After inclusion, women randomized to proactive support of labour will stay at the hospital and have one-to-one midwife support. If no progression during the next 1–2 hours, amniotomy and/or oxytocin stimulation will be started. The control group will adhere to the standard procedures for labour support: expectance until established regular contractions and 4–5 cm cervical dilatation, and then one-to-one midwife support.

**Discussion:**

The idea of proactive support of labour is to initiate early intervention when there are signs of slow progress in order to avoid protracted labour with exhaustion of the mother, the uterus and prolonged stress of the foetus. Proactive support of labour may represent a useful method to improve labour support in nulliparous women. However, evidence based on randomized controlled trials are needed in order to know whether proactive support of labour is comparable or superior to standard care. A randomized, controlled trial is described; challenges and possible clinical implications are discussed.

**Trial registration:**

The Proactive Support of Labor Study (PAF) ClinicalTrials, NCT03056313. Registered on February 17, 2017.

## Background

Poor progression of labour is associated with increased complication rates, such as instrumental delivery, caesarean section (CS) and postpartum haemorrhage [1]. For some women, traumatic birth experience leads to posttraumatic stress disorder [2], and poor birth experience may have long-term influences on the future health of both the woman and her family [3]. Dissatisfaction with birth experience may affect women’s emotional well-being and may even affect the desire to become pregnant again [4].

There is an increasing rate of caesarean sections, reflecting an international trend [5].

In Norway, about 8% of those classified in Robson Group 1 are delivered by emergency caesarean section. Robson group 1 is defined as: nulliparous women with singleton fetuses in cephalic presentation, and spontaneous start of labour at term; it represents one third of all women in labour [6].

Preventing prolonged labour in nulliparous women is important, both for the mother and for foetus [7]. For the foetus, there is an increased risk of asphyxia [8]. For the mother, prolonged labour may be an indication for emergency CS, which in nulliparous women, is the most frequent indication for emergency CS [9]. Prolonged labour is an important cause of fear of childbirth and demand for caesarean section in the next pregnancy. Of all the CSs on maternal demand in the second pregnancy, 70% were due to traumatic birth experience in the first pregnancy [10].

The onset of the active phase of labour is defined as 4–5 cm cervical dilatation and regular contractions [11]. From the active phase, the midwife must ensure progression of labour and take actions (pain relief, amniotomy or stimulation of contractions with oxytocin) if progression is not sufficient [12].

Proactive support of labour was developed by Reuwer et al., in the Netherlands, with the purpose of preventing exhausting and prolonged labour and to facilitate a good birth experience in healthy nulliparous women [7]. Proactive support of labour focuses on the early stage of labour, the latent phase. It defines the start of the active phase of labour as 1–3 cm cervix dilatation, fully effaced cervix (< 5 mm) and painful contractions. Progress in labour is expected at this time, 1 cm per hour, according to Friedman’s curve [13]. Slow progress in the first 3 hours is a clear indication of ineffective contractions. If there is no progression during the next 1–2 hours, stimulation of the contractions should be started first with amniotomy and followed by oxytocin stimulation administered intravenously, after 1–2 hours, if regular contraction are lacking. The idea behind proactive support of labour is that intervention should be initiated early, in latent phase, (before 4 cm dilatation and regular contractions) when there is indication of slow progress. High-quality labour support includes a clear diagnosis of labour and early detection and correction of dysfunctional labour [7].

Proactive support of labour requires good communication throughout the entire process; it includes pre-labour education, psychological support, continuous personal attention and commitment. One-to-one continuous midwife support is already mandatory in early labour [7].

.Nulliparous and multiparous women have different prognoses in terms of birth progression and risk of operative birth [14]. Reuwer et al. emphasize the fundamental differences between the first and subsequent deliveries [7]. A prolonged latent phase in nulliparous women is caused by inefficient uterine contractions in about 10% of cases. Interventions have better effect in early labour, before the mother, uterus and fetus are exhausted [5]. According to Reuwer et al., labour is hard work and should take only one “working day” [7].

Today, labour support is similar in nulliparous and multiparous women. Most women in the latent phase are sent home for expectance and asked to contact the ward when the contractions increase. Active labour is defined as regular contractions and cervical dilatation of 4–5 cm [12].

### This study’s contribution to the field

This study is the first randomized controlled trial (RCT) to compare proactive labour support to standard labour support. It will shed light on the treatment of the latent phase during labour and whether proactive labour support can prevent protracted labour and result in more uncomplicated, normal deliveries.

Here we present the study design and a description of the intervention, as well as provide a detailed outline of how the intervention was planned and implemented at a delivery department in a university hospital.

### Study objectives

The main objective of this study is to examine the potential difference between proactive support of labour compared to current standard labour support, measured as number of uncomplicated, normal vaginal deliveries, and the birth experience of the mothers, as measured by the Childbirth Experience Questionnaire (CEQ) [15].

### Study hypotheses

H^0^: Women randomized to proactive labour support versus those randomized to standard labour support have similar rate of normal deliveries.

H^0^: Women randomized to proactive labour support versus those randomized to standard labour support have no difference in birth experience.

H^1^: Women randomized to proactive labour support have a higher rate of normal deliveries compared to those in the standard labour support group.

H^2^: Women randomized to proactive labour support have better birth experience compared to those in the standard labour support group.

### Study design

The present study is an open-label RCT with randomization to proactive support or standard support during labour. Women will be informed about the study at gestational week 18, and reminded at week 36/37, at an outpatient consultation. Women who meet the inclusion criteria will be included in the study and randomized at arrival to the delivery department.

## Methods/Design

### Study setting

The study takes place at St. Olav’s University Hospital in Trondheim. St. Olav’s Hospital is a public hospital, accepting 3800–4000 women in labour per year. Approximately 1500 of these women are nulliparous. In Norway, antenatal care, delivery care and postpartum care are free of charge.

Since October 2016, nulliparous women attending routine ultrasound screening, gestational week 17–19, have received written information about the study. Recruitment of participants started in March 2017. All health personnel at the delivery ward have been repeatedly informed and reminded about the study both in advance of study start and during ongoing recruitment to the study (Table 1).

**Table 1:** Schedule for enrolment, interventions and assessment

Study period					
	Gestational week 17–19	Gestational week 36	At admission delivery dept.	During labour	6–8 weeks postpartum
Written information	x	x			
Enrolment, obtaining consent			x		
Randomization			x		
Intervention				x	
CEQ (questionnaire)					x

### Study population

Inclusion criteria are women in Robson group 1, planned for vaginal delivery. Robson group 1 represents women who are nulliparous, at term, with spontaneous labour and one foetus in cephalic presentation [9]. The pregnant women and their foetuses are healthy and in such a condition that pending treatment, i.e. return home with contractions, is safe.

Exclusion criteria are women in other Robson groups, insulin-treated diabetes, pre-eclampsia, known uterine malformation (didelphys uterus, septum in uterus, bicorn uterus)**,** other serious illnesses, such as lupus, mother’s heart disease, conditions requiring treatment during pregnancy. Exclusion criteria are also severe conditions in the foetus, such as development abnormalities, pathological Doppler, Rh-immunization, and other conditions that require extra monitoring and that prevent the midwife from practicing a pending attitude.

### Interventions

The participants in the intervention group (proactive) are defined as being in active labour. They stay at the delivery ward and receive one-to-one midwife support. A study flow chart guides the follow-up of the participant (Fig. 2). Vaginal assessment of the cervix status is performed hourly, until 3 cm dilatation of the cervix. If no progression after 1 hour, amniotomy is performed to stimulate contractions. If there is no progression, stimulation of contractions with oxytocin intravenously is initiated. We follow St. Olav’s Hospital’s standard procedure of labour management for use of oxytocin for stimulation in labour (i.e. a dosage of oxytocin, starting with a low dose). The contractions’ duration, strength and frequency are continuously assessed to prevent overstimulation.

The control group follows the standard routine at the delivery department. The women in the control group are defined as being in the latent phase. Usually, they return home to await the progression of labour and are asked to come back when they feel the need for a new assessment. They are defined as being in active labour once they reach 4–5 cm dilatation of the cervix and with regular contractions. The midwife then follows standard procedures of labour management at the delivery department and provides one-to-one support. Vaginal assessment is conducted when needed or, at the latest, after 4 hours. If the assessment shows no sign of progression of labour, amniotomy and oxytocin stimulation is initiated according to the procedures at St. Olav’s Hospital. Women in both study groups are allowed to have various pain relief treatments, for example baths, warm bags, acupuncture and epidural analgesics. The type, dosage and duration of pain relief is registered.

Women who choose not to participate in the study are treated according to standard procedures. They usually return home to wait for progression of the labour.

Normal delivery in the present study is defined as a spontaneous vaginal birth with bleeding less than 500 ml, no fever, no shoulder dystocia and no vaginal tear grade III or IV, and Apgar =/> 7 after 5 minutes. “Pathological delivery” is defined as a delivery that does not meet the criteria for “normal delivery”.

Birth experience will be evaluated by the “Childbirth Experience Questionnaire” (CEQ). The questionnaire, developed in Sweden, is to assess different aspects of first-time mothers’ childbirth experience. The questionnaire contains four dimensions: own capacity, professional support, perceived safety and participation. Multi-trait scaling analysis confirmed the fit of the model [15].

### Primary outcome

The primary outcomes of the present study are (1) the rate of normal deliveries and (2) the birth experience measured by the CEQ questionnaire.

### Secondary outcomes

The secondary outcomes of this study are (1) the duration of labour, in hours and minutes, (2) the number of operative deliveries (vacuum/forceps or caesarean sections), (3) use of pain relief such as epidural analgesic, (4) the use and the duration/dosage of oxytocin during labour, and (5) number of vaginal examinations provided by the midwife or gynaecologist.

### Assignment of interventions and collection

Written information with a consent form is given to all nulliparous women during pregnancy. When they have signed consent, these women may be included in the study at birth if they fulfil inclusion criteria.

Randomization is open, (not blinded). Randomization is computer-based, supported from the Unit for Applied Clinical Research at the Norwegian University of Science and Technology (NTNU). At randomization, the women are stratified according to body mass index (BMI), because obesity influences almost all aspects of pregnancy and labour. The groups are stratified according to first trimester BMI: BMI < 25, BMI 25–30 or BMI > 30.

### Collection of data

**Fig. 1:**
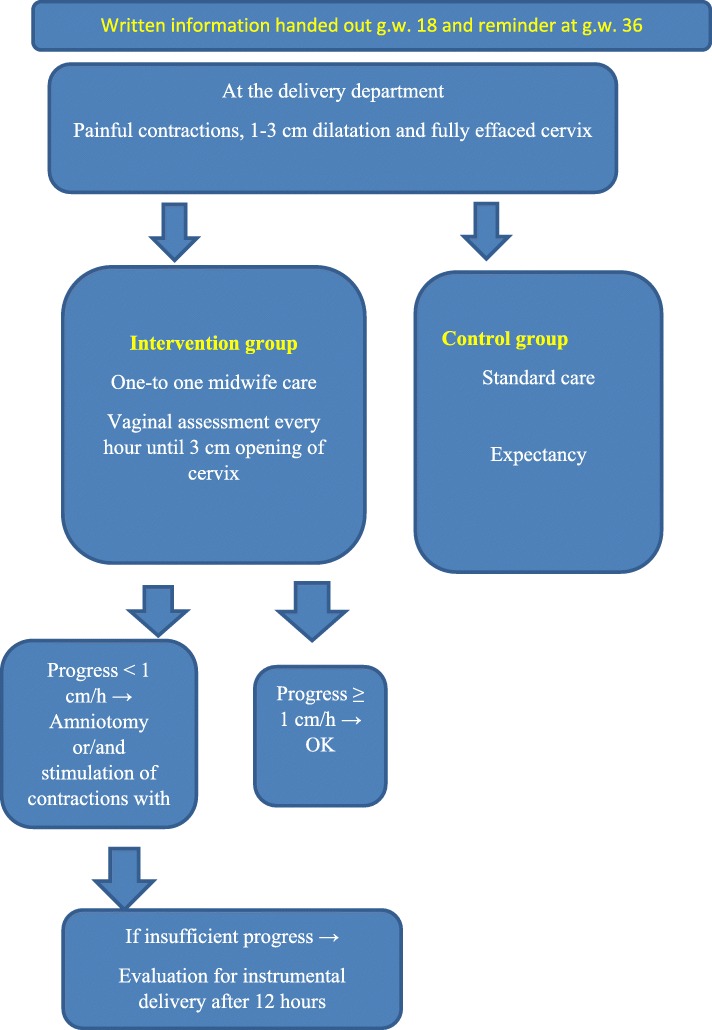
Flow chart for midwives in the delivery department. *g.w*. gestational week

**Fig. 2:**
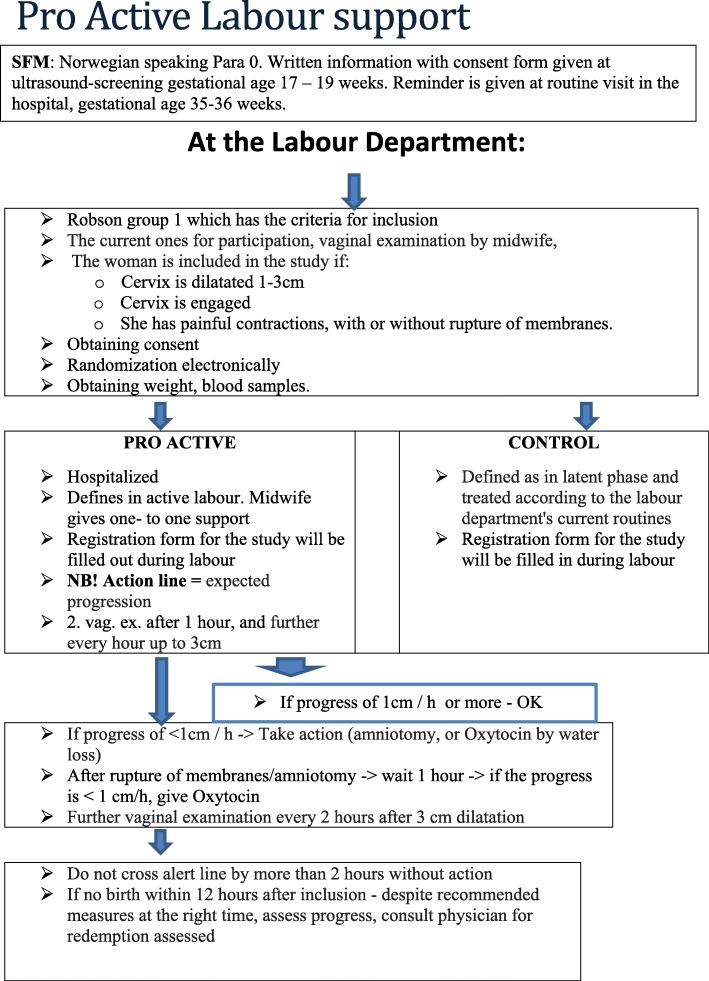
Flow chart for midwives in the delivery department, in details edition

A flow chart (Figs. 1 and 2) guides the midwives through different procedures, before and during delivery and follow-up. Blood samples are collected for later analyses of hormones and cytokines to explore them as risk markers/indicators for prolonged labour. They will be centrifuged and stored in a freezer at −80 degrees.

According to the flow chart, after 12 hours, the midwife will, together with the gynaecologist on call, evaluate instrumental delivery if there is insufficient progress. According to Reuwer et al., if the flow chart is followed, this will rarely be necessary [7].

The midwife who is responsible for the woman in labour collects written consent upon arrival. In Norway, all pregnant women have a health card for follow-up throughout their pregnancy. The health card is collected upon arrival at the hospital. The documentation during labour and postpartum is performed by an electronic medical file system (Doculive: Cerner Sverige AS company EPJ© and Natus: Natus Medical Incorporated ©). For the participants in the present study, the midwife will additionally report study-specific data in a structured form.

The project coordinator midwife will collect the data from the study form, the health card and from the participants’ medical files. A case report form (CRF) has been prepared in cooperation with the Unit for Applied Clinical Research at the NTNU. All data relevant for the study will be registered in the CRF. The CRF is electronic and web-based.

Maternal satisfaction with the birth experience will be assessed by the validated CEQ questionnaire. This questionnaire will be mailed to the mothers 6–8 weeks after birth. The mothers will be asked to return the form in a post-paid envelope.

### Statistical analyses

#### Power calculation

There are around 4000 deliveries per year at St Olav’s University Hospital of Trondheim. Approximately 1500 are nulliparous women. Among these, 10% are estimated to have protracted delivery. About 150–200 nulliparous women per year are expected to meet the inclusion criteria. We assume that the proportion of pathological births in this group is about 30%. To detect a 50% reduction in pathological births in the proactive group with 80% power, we will need 120 women in each group (http://clincalc.com/stats/samplesize.aspx).

##### Study variables

Baseline variables are: (1) age, (2) civil status, (3) education and (4) former gynecological history (miscarriage, extra uterine pregnancy).

Variables reported during labour are: (1) number of vaginal examinations, (2) use and dosage of oxytocin before the baby is born, (3) use of spinal/epidural analgesics, (4) episiotomy, (5) mode of delivery, (6) haemorrhage, (7) perineal lacerations and (8) duration of labour.

New-born variables are: (1) weight, length and head circumference, (2) Apgar score at 5 and 10 minutes and (3) number of transmissions to neonatal intensive care unit.

The CEQ includes 22 items formulated as positive and negative statements. Each item will provide one variable in the data collection. The response format has a 4-point Likert scale, ranging from 1– totally agrees, 2 – mostly agree, 3 – mostly disagree, 4 – totally disagree. Memory of labour pain, sense of security and control are assessed with visual analogue scale (VAS). The VAS scores will be transformed to categorical values. Each item will provide one variable in the data collection [15].

No interim analyses are planned. Both types of management of labour are practiced in Norway.

##### Data Monitoring Committee

Independent of the investigators and midwives who include participants, there will be one obstetrician and one midwife, both experienced and clinically active who will monitor the study. As a first step, they will monitor (1) eligibility to the study and (2) protocol adherence blinded for primary outcome, second, when this is done, they will monitor the primary outcomes.

##### Statistical analyses of data

We will report data both as intention to treat and as per protocol. Per protocol data results will be considered as the clinically relevant, as the study is based on two different protocols for management of labour. The statistical program IBM SPSS Statistics for Windows version 25 will be used (IBM Corp., Armonk, NY, USA). We will report missing data as missing data. No imputation will be made.

Differences between categorical variables will be analysed by Pearson chi-square test.

*P* values < 0.05 are considered statistically significant. Continuous variables will be analysed by odds ratio and standard mean differences. Confidence intervals will be reported.

##### Ethical considerations

The present study is approved by the Regional Committee of Ethics in Medical Research (REK), Central Norway (2014/1788/REK midt), NUST.

Study participation is voluntary. Written information is provided during pregnancy before onset of labour. The possible participants may contact the study midwives by e-mail, if they have additional questions.

All participants are assigned an identification number and letters. These are treated anonymously in all analyses. The two project coordinator midwives and the principal investigators (RD, EV) have access to the consent forms and data collection documents. The documents and electronic documentation with names and personal identification numbers are stored securely in locked cabinets. Potential protocol modifications are reported to REC and continuous communication between project coordinator and the midwives enrolling participants to the study, is assured.

All patients in Norway have public insurance that covers patient injuries caused by the public health and care services, this includes participating in approved clinical studies.

The results are intended to be published in international, peer reviewed journals and disseminated at relevant conferences and meetings.

We will give access to protocols and data on relevant request and on basis of cooperation, after the results are published.

## Discussion

This protocol describes an intervention study comparing two different methods of the treatment of the latent phase during labour. Standard management of labour is compared to proactive management of labour, which is early intervention according to a defined protocol, managing women in Robson group 1. Design, sample, primary and secondary outcomes and statistical methods are presented. The protocol is conducted in accordance with Standard Protocol Items: Recommendations for Interventional Trials (SPIRIT) 2013 statement for clinical trial protocol (Additional file 1). The study is performed in accordance with principles of Good Clinical Practice [16].

The overall aim of the study is to explore whether proactive support of labour can increase the number of normal deliveries in nulliparous women compared to standard support of labour.

### Timing of information about the study

The REK required that written information must be given to the participants before they are in labour. Participants can unsubscribe from the program at any time and without giving reasons.

Written information is therefore given to all nulliparous pregnant women at the ultrasound-screening and consultation in pregnancy week 18. Our experience is that this time point is too early in pregnancy, and many women forget about the study when entering the hospital with possible labour. Accordingly, since March 2018 we are repeating the information about the study at a routine visit for nulliparous women at the hospital in week 36/37. At this time point, they are more receptive to a study about labour support.

### Repeated information to colleagues

Evaluation after 1 year of inclusions has revealed major challenges that needed to be addressed. Dialogue with the midwives in the maternity ward revealed a gatekeeper effect due to midwives’ limited motivation to recruit, and include, women to the study. The midwives’ questions and concerns were collected and reviewed thoroughly by the research group. Concerns and limited motivation were due to the fear of unnecessary interventions such as amniotomy, oxytocin use and an overall aim of a “natural delivery”. Midwives in the delivery department were concerned that the evaluation after 12 hours for instrumental delivery might lead to an increased rate of caesarean section. A subsequent explanatory information letter was distributed individually to all midwives. In addition, the questions and concerns were discussed with the midwife group. The written form and the flow chart were adjusted to avoid misinterpretations. Consequently, more women were included in the study.

Concerns about lack of evidence-based practice, were not expressed. Motivation in the midwife group increased with improved information and dialogue. The quality of the study is dependent on the midwives’ compliance to the flow chart during labour for the intervention group.

### Blinding

Blinding is not possible in such a RCT-study. A spillover effect might be expected. Midwives care for women in both the intervention group and in the control group. This might affect the result, based on the midwife’s experiences when she follows the two different procedures. We realize that it might be a challenge for the midwives to follow up labour according to two different “protocols”. The spillover effect is expected to be limited and not expected to reduce the validity of the study.

The standard care depends on the capacity at the delivery department, the midwife’s assessment and the woman’s capability to ask for what she needs. Women randomized to the control group are not always asked to return home if they are exhausted or live far away. These women might stay in the ward and are especially prone to a spillover effect. This might mask differences between standard care and the intervention care.

### Continuous midwife support

According to previous research, women with continuous support during childbirth are more likely to have a spontaneous vaginal birth, less likely to have intrapartum analgesics and less likely to report dissatisfaction [14].

In 2015, at St. Olav’s Hospital, a clinical study in the delivery department explored the midwife’s presence on the labour room during active labour. This study found higher maternal satisfaction with the midwife’s presence, and the findings changed the way midwives work at the department. It is now more common that the midwife stays in the labour room during the entire active labour and gives one-to-one support. This is one of the important principles for the proactive support of labour.

The strengths of this present study are many, particularly the randomized, controlled clinical study design. This design minimizes the chance for confounding. The women are also stratified according to BMI to minimize the risk of uneven randomization. RCT is the gold standard for studying causality, which is the objective of this study.

### Trial status

The study is currently recruiting participants. Since study start in April 2017 and through May 2019, 90 eligible women have been included in the study. Two women were excluded due to undetected breech presentation and one due to assumption of a vasa previa. Two were included on the wrong premises. Four were randomized to the intervention group, but the midwife did not follow the protocol. One woman wanted to leave the study after inclusion. The estimated time for end of inclusion will be December 2020.

## Conclusions

Critical review of procedures and methods is expected from health professionals and scientists. The premises for pregnancy and labour support have been extensively altered during the last 60 years. Critical evaluation of present labour support methods is necessary and labour support should be based on evidence. Both labour support methods described in the present study are currently in use. It is ethically justified and imperative to perform randomized studies to evaluate obstetric management.

## Supplementary information

**Additional file 1.** SPIRIT 2013 checklist: recommended items to address in a clinical trial protocol and related documents

## Data Availability

Not applicable.

## Abbreviations

BMI: Body mass index
CEQ: Childbirth Experience Questionarie
CRF: Case report form
CS: Caesarean section
G.w: Gestational week
NTNU: Norwegian University of Science and Technology
RCT: Randomized controlled trial
REK: Regional Committee of Ethics in Medical Research
VAS: Visual analogue scale
